# Integrated analysis miRNA and mRNA profiling in patients with severe oligozoospermia reveals miR-34c-3p downregulates PLCXD3 expression

**DOI:** 10.18632/oncotarget.10947

**Published:** 2016-07-29

**Authors:** Zhiming Li, Zaozao Zheng, Jun Ruan, Zhi Li, Xuan Zhuang, Chi-Meng Tzeng

**Affiliations:** ^1^ Translational Medicine Research Center (TMRC), School of Pharmaceutical Sciences, Xiamen University, Xiamen, Fujian, China; ^2^ Key Laboratory for Cancer T-Cell Theranostics and Clinical Translation (CTCTCT), Xiamen University, Xiamen, Fujian, China; ^3^ INNOVA Cell Theranostics/Clinics and TRANSLA Health Group, Xiamen University, Xiamen, Fujian, China; ^4^ Department of Urology, The First Affiliated Hospital of Xiamen University, Xiamen, Fujian, China

**Keywords:** global miRNA and mRNA profile, integrated analysis, miR-34c-3p, PLCXD3, spermatogenesis, Pathology Section

## Abstract

Our previous research suggested that an integrated analysis of microRNA (miRNA) and messenger RNA (mRNA) expression is helpful to explore miRNA-mRNA interactions and to uncover the molecular mechanisms of male infertility. In this study, microarrays were used to compare the differences in the miRNA and mRNA expression profiles in the testicular tissues of severe oligozoospermia (SO) patients with obstructive azoospermia (OA) controls with normal spermatogenesis. Four miRNAs (miR-1246, miR-375, miR-410, and miR-758) and six mRNAs (SLC1A3, PRKAR2B, HYDIN, WDR65, PRDX1, and ADATMS5) were selected to validate the microarray data using quantitative real-time PCR. Using statistical calculations and bioinformatics predictions, we identified 33 differentially expressed miRNAs and 1,239 differentially expressed mRNAs, among which one potential miRNA-target gene pair, miR-34c-3p and PLCXD3 (Phosphatidylinositol-Specific Phospholipase C, X Domain Containing 3), was identified. Immunohistochemical analysis indicated that PLCXD3 was located within the germ cells of the mouse and human testis. Moreover, we found that miR-34c-3p was able to decrease PLCXD3 expression in mouse (GC-1 and TM4) and human (NCM460) cell lines, presumably indicating the possibility that miR-34c-3p acts as an intracellular mediator in germinal lineage differentiation. Notably, we reported the expression of the PLCXD3 protein in a man with normal spermatogenesis and the lack of the PLCXD3 protein in a man with SO. Therefore, the identified miRNA and mRNA may represent a potentially novel molecular regulatory network and therapeutic targets for the study or treatment of SO, which might provide a better understanding of the molecular basis of spermatogenesis dysfunction.

## INTRODUCTION

Infertility is global health problem, and the increasing trend in infertility deserves more scientific attention. Approximately 15% of couples experience infertility, and 75% of the cases are idiopathic in which the molecular mechanisms underlying the defects remain unknown. Male factor infertility is estimated to account for 50% of infertility cases, and a significant proportion of male infertility is accompanied by idiopathic azoospermia (a zero sperm count) or severe oligozoospermia (sperm count ≤ 5 million/mL) [1]. Although there is no consensus definition of severe oligozoospermia (SO), a sperm concentration of 5 × 10^6^/mL was chosen as the detection limit for case selection, based on a detailed review of the recent literature on the evaluation of male fertility [2-4]. It is recognized that men with very low sperm counts have a higher incidence of Y-chromosome microdeletions (up to 17%) [5]. However, the genetic causalities of most cases of low sperm count have not been elucidated.

To date, studies using knockout mouse models have recently linked many genes to spermatogenesis, and the mechanisms of these genes are currently being clarified. These animal findings have yet to be shown as applicable to most human cases because the knockout mouse phenotype is not always faithfully reproduced in humans. Microarray technologies have been successfully used to identify biomarkers, drug targets, and mechanisms of disease subtypes. Microarrays are valuable tools for the identification of gene expression profiles of infertile phenotypes. Transcriptome analyses could be used to determine differentially transcribed genes and to enhance our understanding of transcriptional regulation during spermatogenesis. Furthermore, evidence suggests that microRNAs (miRNAs) have critical functions in mammalian spermatogenesis and play important roles in regulating post-transcriptional gene silencing *via* base pair binding to the 3′ untranslated regions (3′-UTR) of their target mRNAs [6]. Therefore, it is reasonable to speculate that aberrant miRNA expression would also be associated with male infertility. A novel comprehensive analysis of the global influence of mRNAs and miRNAs may lead to further developments in the understanding of the etiology of male SO through the identification of specific infertile phenotype signatures in the testis [7].

A simple method was previously created by our team and was used to identify promising novel correlation signatures of these miRNA/mRNA pairs and dysfunctional biological processes [8]. However, no studies have examined the global miRNA and mRNA expression profiles in SO testis tissue compared with normal tissues. In this study, we first applied microarrays to integrate the miRNA and mRNA expression profiles of testis specimens from SO patients and from subjects with obstructive azoospermia (OA) (a histological examination showed normal spermatogenesis) to identify the specific genes, miRNAs and molecular pathways responsible for SO. We then validated the markedly altered miRNAs and genes in the same cohort. Subsequently, we discovered and constructed a post-transcriptional regulatory network with the miRNA-target gene pair miR-34c-3p and PLCXD3 (Phosphatidylinositol-Specific Phospholipase C, X Domain Containing 3) to shed light on the interplay between mRNAs and miRNAs. The correlation between the expression levels of miR-34c-3p and PLCXD3 provides valuable data for the analysis of spermatogenic dysfunction in male infertility. The identified miRNA and mRNA may represent a potentially novel marker and molecular regulatory network, which may provide a better understanding of the molecular basis of spermatogenesis.

## RESULTS

The karyotype and Y-chromosome analyses of the current subjects were normal (46XY) and did not show microdeletions. Furthermore, with the aim of elucidating the molecular mechanisms underlying SO at the transcriptome level, we first compared the miRNA-mRNA profiles of SO to those of OA using microarrays and discovered the direct mRNA targets of the miRNAs based on the anti-correlation data of the differentially expressed miRNAs and mRNAs.

### Differentially expressed genes, miRNAs and cluster analysis

**Figure 1:**
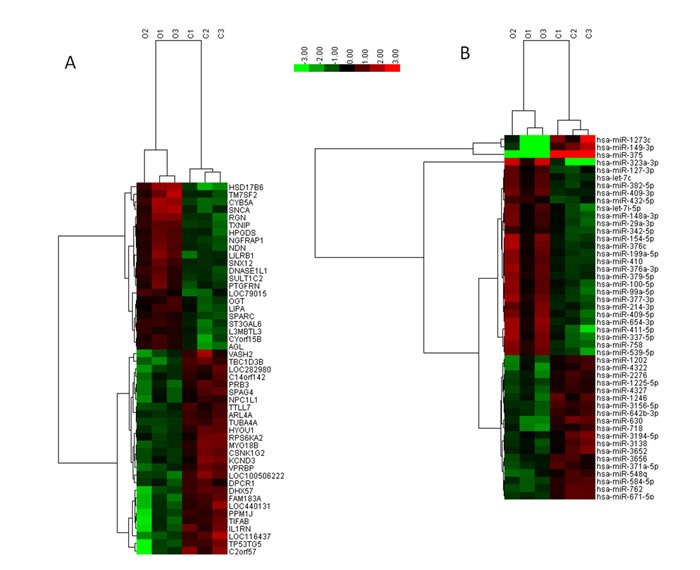
Hierarchical clustering of mRNAs A. and miRNAs B. in testicular tissue samples Testicular tissue samples were clustered according to the expression profiles of 49 differentially expressed mRNAs with a two-fold change in expression (**A**) and 33 differentially expressed miRNAs with a two-fold change in expression (**B**) between SO patients (*n* = 3) and OA (*n* = 3) patients. All samples were properly assigned to the correct class. The key color bar indicates that the mRNA and miRNA expression levels increased from green to red compared to the OA samples. A dark color indicates that the expression levels in the two groups were similar. C: Obstructive-azoospermia controls; O: Severe oligozoospermia.

We first obtained testis tissue samples from infertile men to study the changes in mRNA and miRNA expression in SO patients compared with OA patients. After applying a stringent filtering approach that compared SO with OA (*p*-value < 0.05, fold change>2 or < 1/2), we identified 560 upregulated genes and 679 downregulated genes, as well as 18 upregulated miRNAs and 15 downregulated miRNA. Based on these differentially expressed miRNAs, a tree with a clear distinction between the SO and OA patients was generated by cluster analysis (Figure 1). The cluster analysis revealed a high degree of similarity within groups and showed that SO patients can be robustly separated from OA patients. Taken together, the results showed that there was a significant difference in the mRNA and miRNA expression profiles between the two groups when we compared the transcriptome data from the SO and OA samples.

### GO enrichment of differentially expressed genes

**Figure 2:**
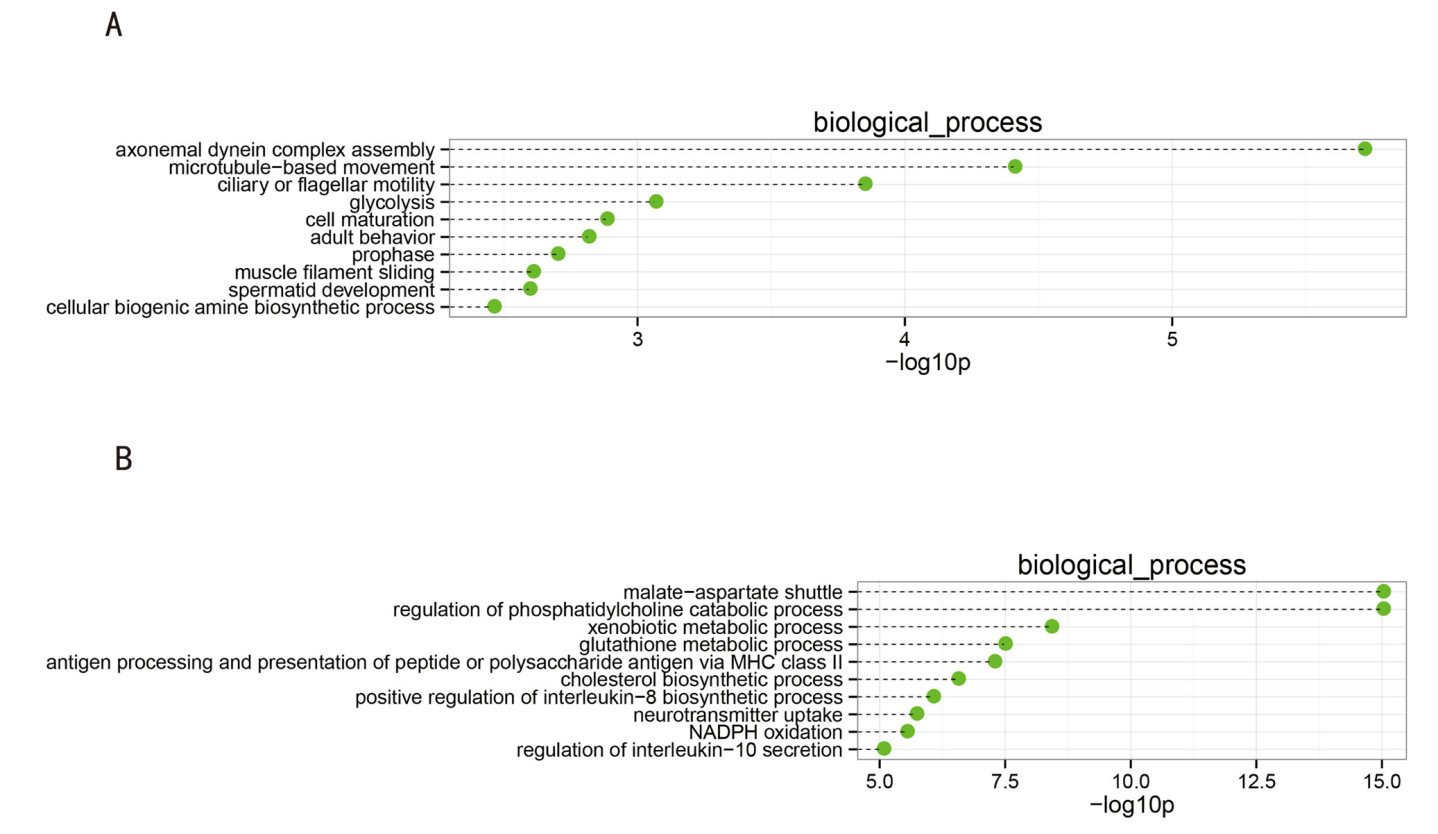
Top 10 categories of the GO biological processes associated with the significantly downregulated (**A**) and upregulated genes (**B**). The dashed lines represent the p-values for the ten top-ranked categories of GO biological processes associated with the downregulated (upper panel) and upregulated (lower panel) genes. The p-values were calculated using hypergeometric tests and corrected using the Benjamini-Hochberg adjustment. The p-values are expressed as negative logarithms (base 10).

We identified the functional categories of these genes by performing a gene ontology (GO) analysis to gain further insights into the biological processes that were potentially mediated by the genes that were differentially expressed in spermatogenesis. For the downregulated genes, 52 GO terms in the category of biological process were significantly enriched at a FDR threshold of < 0.05. As shown in Figure 2A, the top 10 significantly enriched biological processes were: axonemal dynein complex assembly, microtubule-based movement, ciliary or flagellar motility, glycolysis, cell maturation, adult behavior, prophase, muscle filament sliding, spermatid development, and cellular biogenic amine biosynthetic process. Many biological processes were involved in functions related to spermatogenesis, such as spermatid development and cell maturation.

There were 192 significantly enriched GO terms (FDR threshold of < 0.05) for the upregulated genes, as shown in Figure 2B. The top 10 significantly enriched biological processes were: malate-aspartate shuttle, regulation of phosphatidylcholine catabolic process, xenobiotic metabolic process, glutathione metabolic process, antigen processing and presentation of peptide or polysaccharide antigen *via* MHC class II, cholesterol biosynthetic process, positive regulation of the interleukin-8 biosynthetic process, neurotransmitter uptake, NADPH oxidation, and regulation of interleukin-10 secretion. The most abundant categories were those related to physiological processes and metabolism, along with oxidative stress, which may induce peroxidative damage in the sperm plasma membrane and DNA.

### KEGG enrichment of the differentially expressed genes

**Figure 3:**
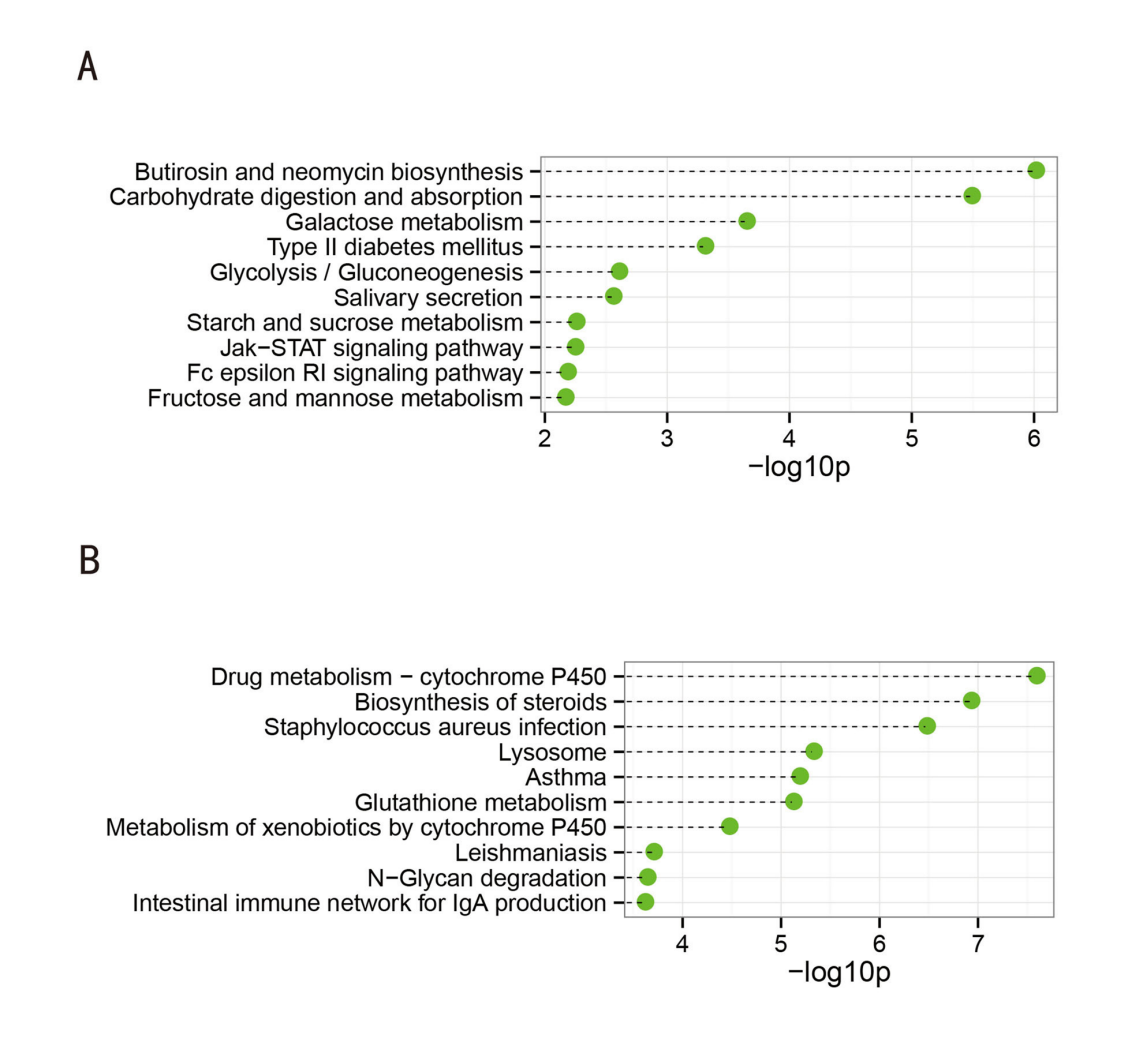
Top 10 categories of KEGG pathways associated with the significantly downregulated (**A**) and upregulated genes (**B**). The dashed lines represent the p-values for the ten top-ranked categories of KEGG pathways associated with the downregulated (upper panel) and upregulated (lower panel) genes. The p-values were calculated using hypergeometric tests and corrected using the Benjamini-Hochberg adjustment. The p-values are expressed as negative logarithms (base 10).

Twenty-four KEGG pathways (FDR *p*-value < 0.05) were associated with the downregulated genes. As shown in Figure 3A, the top 10 significantly enriched biological processes were: butirosin and neomycin biosynthesis, carbohydrate digestion and absorption, galactose metabolism, type II diabetes mellitus, glycolysis/gluconeogenesis, salivary secretion, starch and sucrose metabolism, Jak-STAT signaling pathway, Fc epsilon RI signaling pathway, and fructose and mannose metabolism. Of particular note, evidence shows that proteins of the Jak-STAT signaling pathway are expressed and active in human sperm [9]. Therefore, defects in this signaling pathway in sperm may have relevance to SO. In addition, fructose is secreted by male seminal vesicles, is commonly used as a biomarker of seminal vesicle function, and has an inverse relationship with the sperm count [10]. Indeed, the enrichment of fructose and mannose metabolism in the downregulated genes corresponded to the decreased fructose levels in OS patients (see Supplemental File 1: Patient information).

Forty KEGG pathways (FDR *p*-value < 0.05) were associated with the upregulated genes, as shown in Figure 3B. The top 10 significantly enriched pathways were: drug metabolism-cytochrome P450, biosynthesis of steroids, *Staphylococcus aureus* infection, lysosome, asthma, glutathione metabolism, metabolism of xenobiotics by cytochrome P450, leishmaniasis, N-Glycan degradation, and intestinal immune network for IgA production. The reactive oxygen species (ROS) produced during the accumulation of lipofuscin inside lysosomes and steroid genesis (testosterone synthesis) in Leydig cells might be responsible for the loss of testicular function [11].

### miRNA enrichment of the differentially expressed genes

Based on an analysis of the online databases (MiRanda, Pictar, TargetScans, Microrna.org, PITA, MicroT, and RNA22), 38 miRNAs and 682 miRNAs were significantly enriched in the downregulated and upregulated genes, respectively. Many miRNAs that were shown to have altered expression in previous studies of male infertility, including hsa-miR-30c-1 [12], hsa-mir-34b [13], mir-371 [14], hsa-mir-29c [13], and miR-361 [15], were also enriched in our study. The top 10 miRNAs are shown in Table 1. As expected, most of the miRNAs were significantly correlated with multiple target genes. We also noticed that a significant number of miRNAs (such as miR-516-3p, miR-744 and miR-506) that had not been previously annotated in male infertility studies were enriched in these gene sets.

**Table 1:** The top 10 enriched regulated miRNAs of the down-regulated genes and up-regulated genes

miRNA	Target number	*P*-value*
down-regulated genes		
hsa-miR-30c-1*	32	0.000544
hsa-miR-34b*	26	0.002652
hsa-miR-516-3p	57	0.002867
hsa-miR-218-1*	23	0.006893
hsa-miR-744*	20	0.007258
hsa-miR-877*	20	0.007628
hsa-miR-125b-2*	25	0.009248
hsa-miR-371	57	0.009509
hsa-miR-29c*	27	0.010885
hsa-miR-361	95	0.012226
up-regulated genes		
hsa-miR-513a-3p	305	0
hsa-miR-590-3p	340	0
hsa-miR-548l	291	0
hsa-miR-548n	339	0
hsa-miR-1303	272	3.33E-16
hsa-miR-548m	283	3.11E-15
hsa-miR-124	283	8.22E-15
hsa-miR-1279	219	8.22E-15
hsa-miR-579	305	8.99E-15
hsa-miR-548h	300	1.24E-14

* : The p-values was calculated by hypergeometric tests, and corrected by Benjamini-Hochberg adjustment

### mRNA-miRNA determination

We collected 8,215,441 promising pairs of miRNAs and their putative mRNA targets from the online databases MiRanda, Pictar, TargetScans, Microrna.org, PITA, microT, and RNA22. We also obtained 4,292 anti-correlated miRNA and genes according to our microarray data (Pearson's correlation coefficient < 0 and adjusted *p*-value < 0.05). Two procedures were used to identify the overlap in 242 coding genes and 154 targeted miRNAs, and Table 2 shows the top 10 differentially expressed miRNA-target gene pairs. Moreover, by carefully examining the negatively correlated expression of the individual miRNAs and target genes, we found that PLCXD3, a phospholipase that hydrolyzes phospholipids into fatty acids, may be a novel target of miR-34c-3p. Notably, the pair shows opposite expression patterns in SO patients.

**Table 2:** The top 10 differently expressed miRNA-target relationships

miRNA	Gene Symbol	miRNA FC	Gene FC	θ*
hsa-miR-34c-3p	PLCXD3	0.399001733	7.359275106	2.9363635
hsa-miR-612	CCL3	0.923756255	1.811858261	1.6737154
hsa-miR-1292	CCL3	0.923756255	1.811858261	1.6737154
hsa-miR-567	CCL3	0.923756255	1.811858261	1.6737154
hsa-miR-1276	CCL3	0.923756255	1.811858261	1.6737154
hsa-miR-1266	CCL3	0.923756255	1.811858261	1.6737154
hsa-miR-146b-3p	CCL3	0.923756255	1.811858261	1.6737154
hsa-miR-548l	CCL3	0.923756255	1.811858261	1.6737154
hsa-miR-548a-5p	CCL3	0.923756255	1.811858261	1.6737154
hsa-miR-548i	CCL3	0.923756255	1.811858261	1.6737154

* : θ = FC1/FC2, FC1 means fold change of significantly expressed genes, and FC2 means fold change of significantly expressed miRNAs. Take the reciprocal of FC, if FC<0.

### Validation of mRNA and miRNA expression

**Figure 4:**
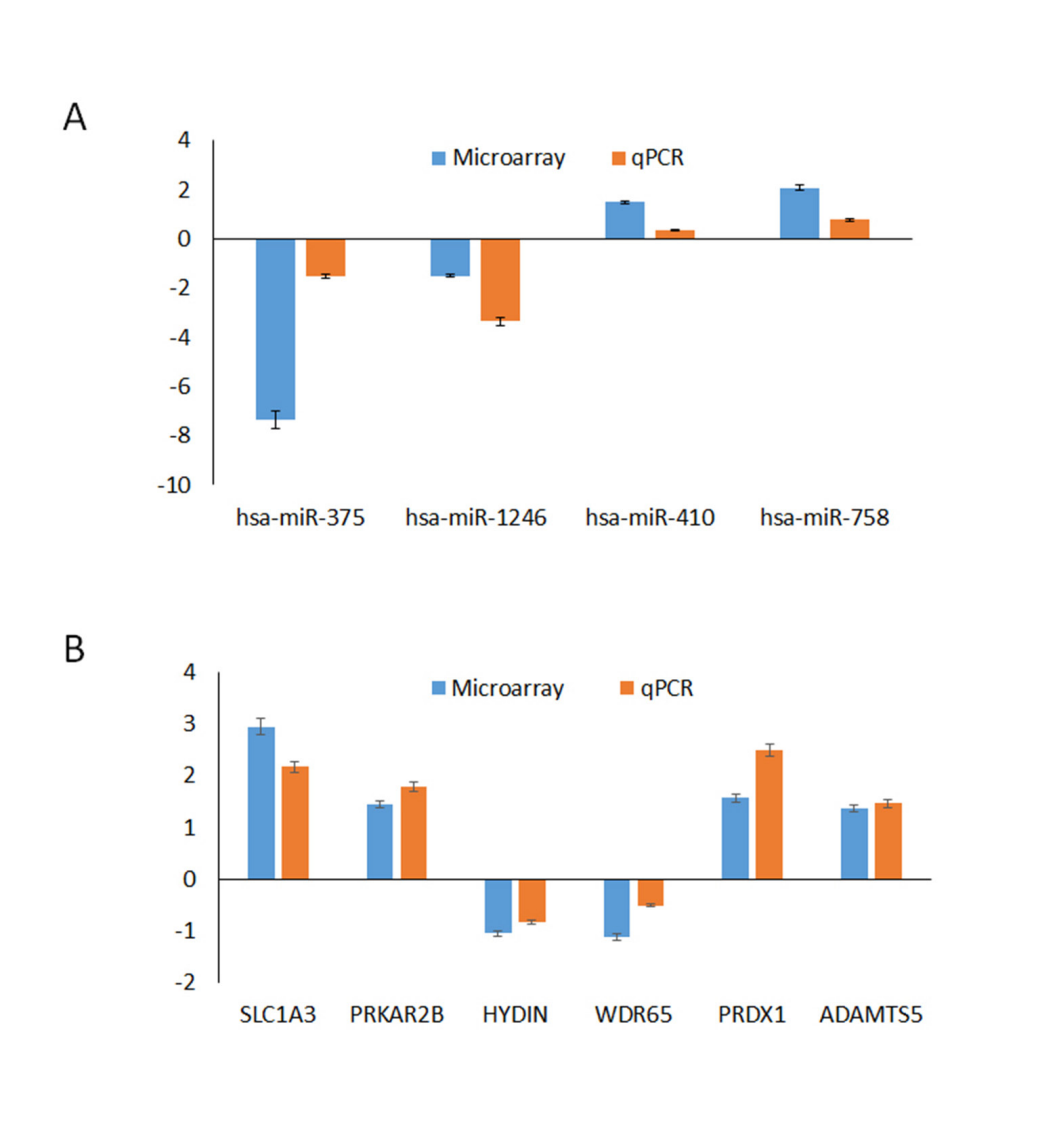
Comparison of the microarray and qPCR results for the mRNAs (**A**) and miRNAs (**B**). Four miRNAs (miR-1246, miR-375, miR-410, and miR-758) and six mRNAs (SLC1A3, PRKAR2B, HYDIN, WDR65, PRDX1, and ADATMS5) were selected to validate our miRNA and mRNA microarray data by qPCR. The height of the columns in the chart represents the log-transformed average fold change in expression for each of the validated genes; the bars represent the standard errors.

Subsets of mRNAs (SLC1A3, PRKAR2B, HYDIN, WDR65, PRDX1, and ADAMTS5) and miRNAs (miR-1246, miR-375, miR-410, and miR-758) that were identified as differentially expressed by the microarray analysis were selected for further validation (Figure 4). RT-qPCR was then performed to assess the expression of these mRNAs and miRNAs in samples from the same testicular tissues. A comparison of the expression levels between the microarray data and the qPCR results showed a strong correlation between the two platforms. The fold changes in the expression of the tested mRNAs and miRNAs determined using RT-qPCR were largely concordant with the microarray data. A melting curve analysis and agarose gel electrophoresis were also used to control for the specificity of the qPCR products. These findings indicated that our miRNA and mRNA microarray data reliably reveal the differences in the transcriptome profile of OS and AO diseases.

### Global microRNA-regulated network integration

**Figure 5:**
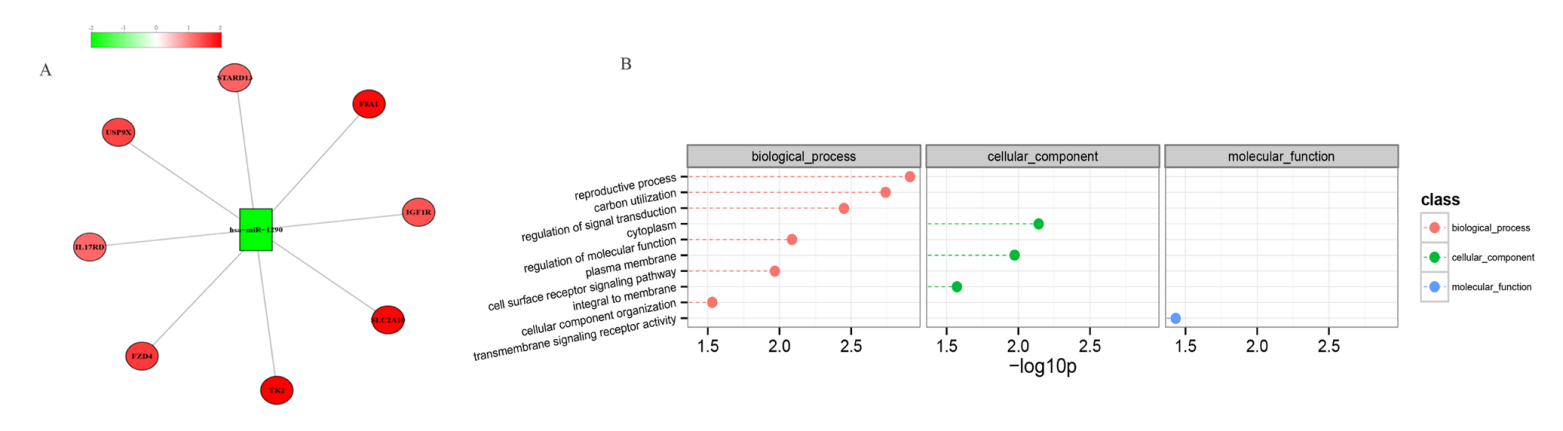
Visualization of a portion of the miRNA-regulated network A. and the involved GO biological processes B Negatively correlated miRNA-mRNA interactions were visualized as a network using Cytoscape. This network gives, for the first time, a theoretical outline of the concerted action of the regulatory miRNAs and their potential target mRNAs in NOA. The red nodes represent the miRNAs, the green nodes represent miRNA targets, and the yellow edges represent the protein-protein interactions (**A**). The dashed lines represent the p-values for the ten top-ranked GO biological processes of the module (**B**). The p-values were calculated using hypergeometric tests and corrected using the Benjamini-Hochberg adjustment. The p-values are expressed as negative logarithms (base 10).

Based on our identified miRNA targets, the experimentally validated miRNA targets in TarBase, and the human protein-protein interaction networks (PPIN), we constructed a global microRNA-regulated network with 2,206 edges (574 miRNA-target and 1,632 PPIN), 791 coding genes, and 165 miRNAs (see Supplemental File 3: network). We exported the MCL-generated connectivity information to Cytoscape and observed the various degrees of miRNA-mRNA interactions to further visualize the miRNA-mRNA interactions. Figure 5 shows one of the observed modules in the global miRNA-regulated networks. Remarkably, we also observed that the target genes (IGF1R, FZD4, USP9X, SLC2A10, TK2, STARD13, F8A1 and IL17RD) and has-miR-1290 sets of this module were implicated in the reproductive process and regulated the signal transduction pathways and molecular functions that reflected the defects in spermatogenesis.

### Association of PLCXD3 with spermatogenesis

**Figure 6:**
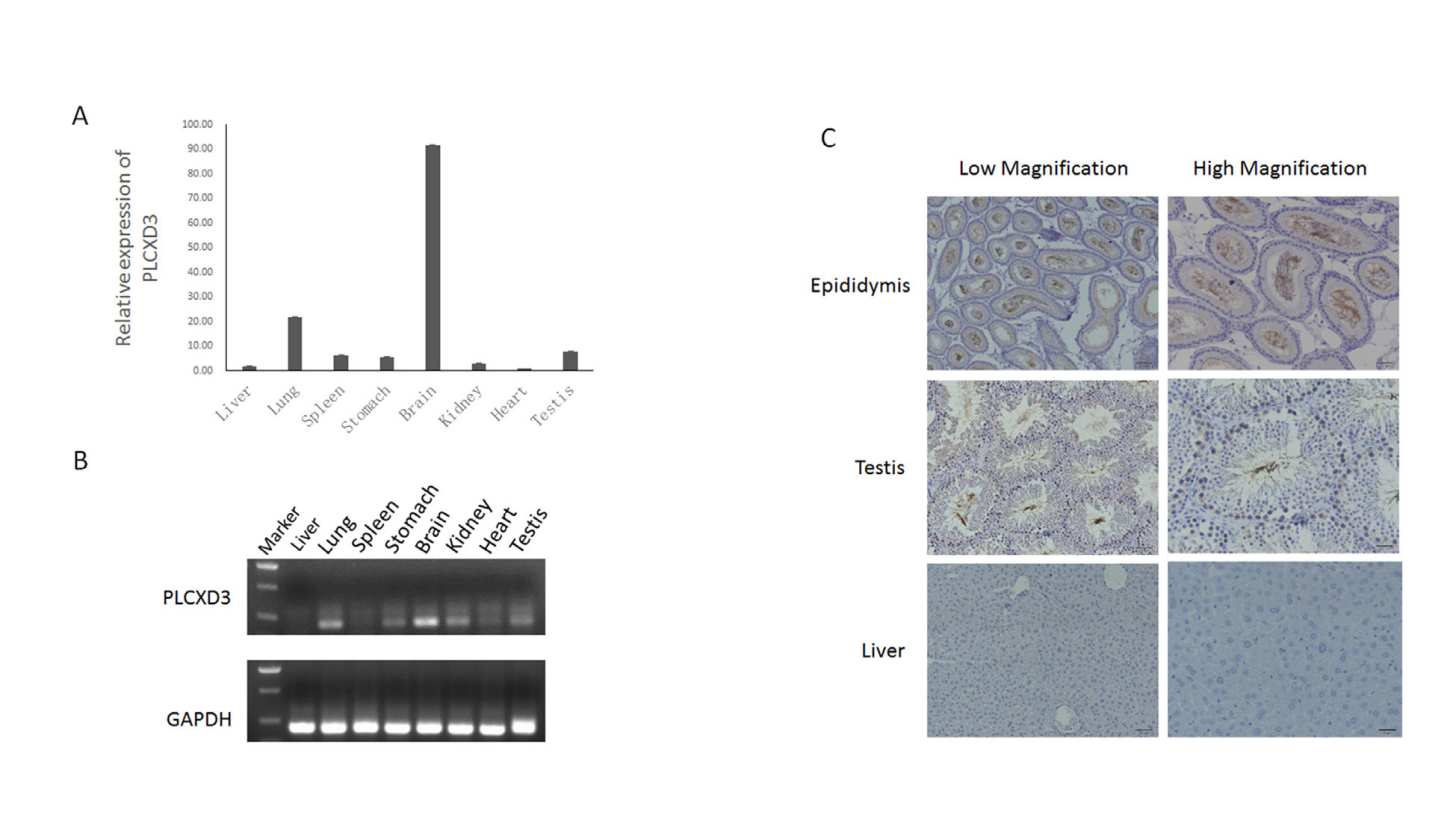
Expression pattern of PLCXD3 in selected mouse tissues **A.** The relative expression levels of the PLCXD3 mRNA in selected mouse tissues were detected by real-time PCR. GAPDH was used as an internal control. The results are expressed as the mean ± SD of three independent experiments. **B.** Semi-quantitative RT-qPCR analysis of the expression levels of the PLCXD3 mRNA in a restricted number of mouse tissues. **C.** Compared with mouse liver tissue, PLCXD3 was upregulated in the mouse epididymis and testis and was detected by immunohistochemistry. Representative staining at different magnifications (200× and 400×) is shown.

PLCXD3 is a member of the PLCXD gene family. The proteins encoded by the gene family are a seventh class of PI-PLC (Phosphatidylinositol-specific phospholipase C) enzymes. PI-PLC is able to mediate the physiological actions of various hormones, growth factors and neurotransmitters by regulating cytosolic calcium levels and/or the activity of several protein kinases [16]. The amino acid sequences of the PLCXD3 protein were highly conserved across species, including human and mouse, suggesting conserved functions [17]. First, quantitative real time PCR was used to determine relative levels of the PLCXD3 mRNA in several mouse tissues (Figure 6A). The qPCR results indicated that the PLCXD3 mRNA had a distinct tissue expression pattern. Of the eight mouse tissues, the highest mRNA expression levels were observed in the brain. PLCXD3 mRNA expression was also observed in the testis, indicating a potential fundamental role in spermatogenesis. The RT-qPCR analysis suggested similar tissue distributions (Figure 6B). The localization of PLCXD3 was investigated using immunohistochemistry. In the epididymis, there is strong signal for PLCXD3 in stereocilia, which, structurally, are long microvilli, and it is likely that PLCXD3 is involved in water (or solute) transport in these cells, which are known to be engaged in substantial water reabsorption during spermiogenesis (Figure 6C). PLCXD3 is also present in spermatogonia, spermatocytes and spermatids in the testis, but its role here remains obscure. Interestingly, a significant lack of expression for the PLCXD3 mRNA and protein was observed in mouse liver. PLCXD3 exhibited tissue-specific expression profiles in mice, and immunocytochemistry revealed distinct sub-cellular localization patterns in several tissues.

### miR-34c-3p downregulates PLCXD3 expression

**Figure 7:**
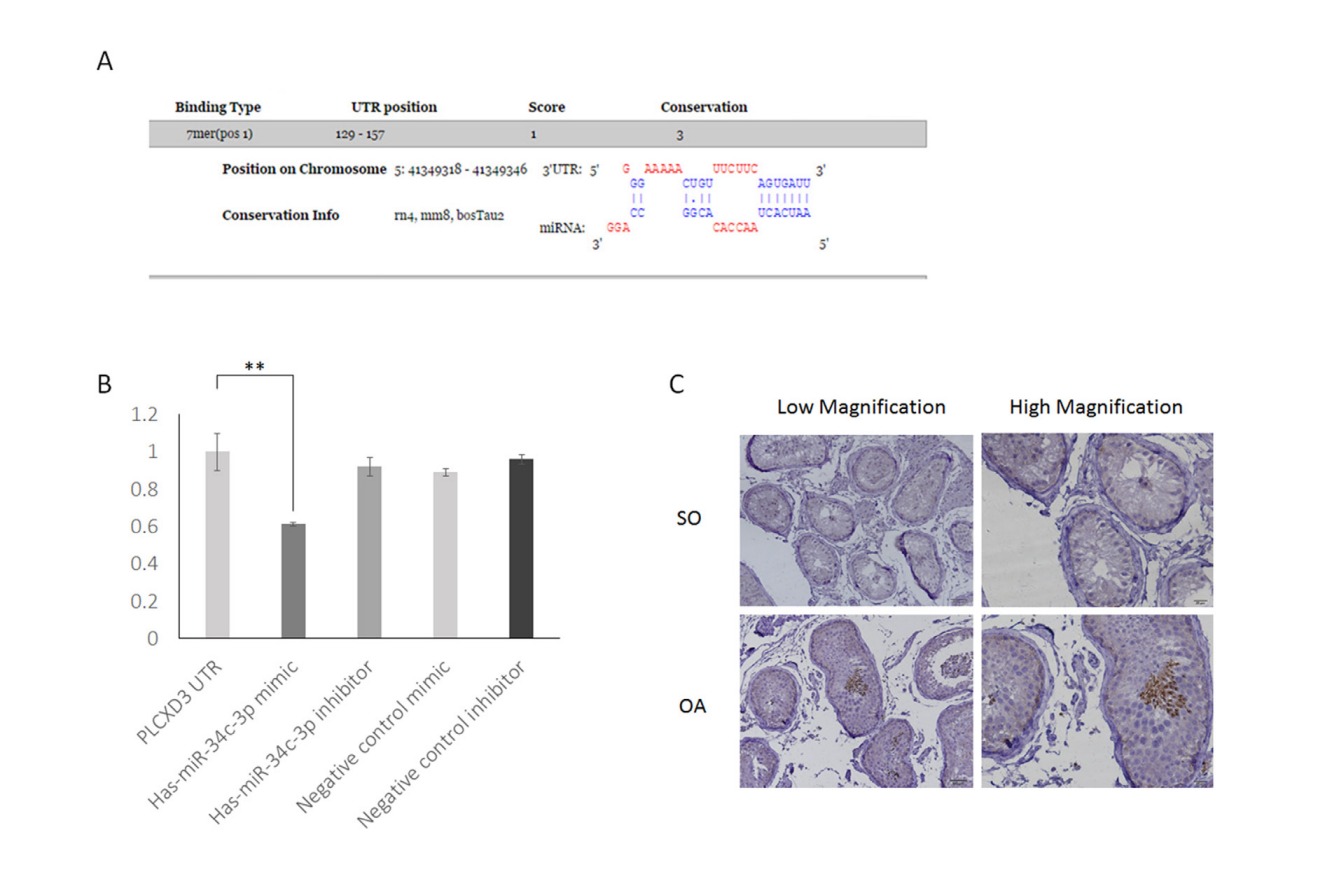
Confirmation of PLCXD3 as the target gene of miR-34c-3p **A.** The PLCXD3 3′-UTR region has a conserved miR-146b-5p-binding site, as predicted by DIANA-microT 3.0. **B.** miR-34c-3p significantly reduced the luciferase activities of the pmirGLO-PLCXD3 reporter compared with the scrambled miRNA negative control (NC). This downregulation was efficiently prevented by the miR-34c-3p inhibitor. The blank vector (pmirGLO-Control) has no seed-binding site, and thus firefly luciferase activity was not affected by miR-34c-3p. **C.** Immunohistochemistry of testis biopsies from men with OA showed a strong positive expression in germ cells at low power 200× and high power 400×, but almost no expression in the SO group. SO: Severe oligozoospermia, OA: Obstructive-azoospermia.

**Figure 8:**
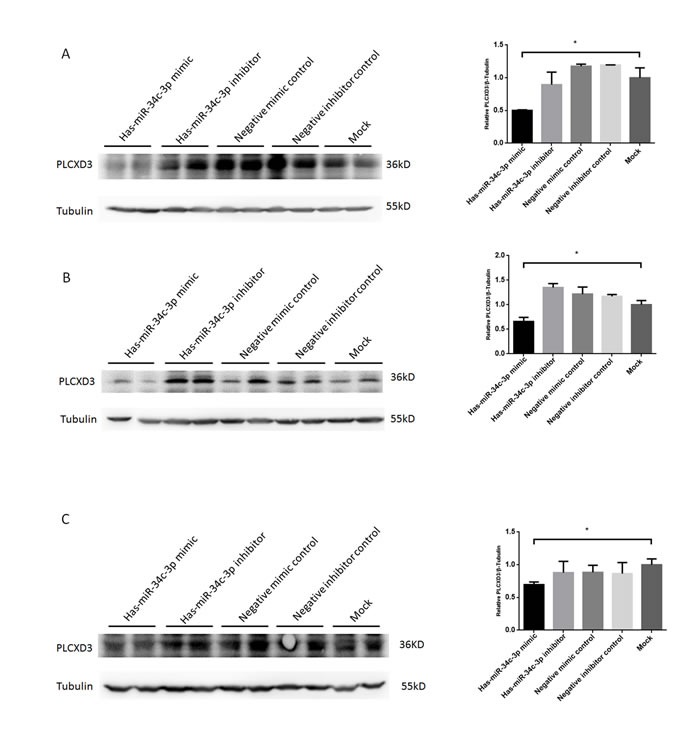
Verification of posttranscriptional repression of PLCXD3 by miR-34c-3p in multiple cell types Mouse spermatogonia GC-1 (**A**), Mouse Sertoli TM4 cells (**B**), and human colonic epithelial NCM460 cells (**C**) were transfected with miR-34c-3p mimic/inhibitor (50 nM, 100 nM, respectively), or negative control mimic/inibitor (50 nM, 100 nM, respectively) and PLCXD3 protein levels assayed 48 h post-transfection by Western blot (two technical replicates). *P < 0.05; **P < 0.01.

Based on the miRNA-mRNA interaction analysis and the results of the PIAT, RNAhybrid, and DIANA predictions, PLCXD3 was a potential target gene of miR-34c-3p (Table 2). We also found that the 3′-UTR region of the PLCXD3 region contains one highly conserved miR-34c-3p-binding site (Figure 7A). We chose this promising pair for experimental validation in the GC-1 and TM-4 mouse cell lines and in the NCM460 human cell line to further assess the validity of the miRNA-mRNA interaction. We performed dual luciferase assays to confirm the potential binding between miR-34c-3p and PLCXD3. The luciferase activity of the PLCXD3 reporter was decreased by 61.2% upon co-transfection with the miR-34c-3p mimic (*P* < 0.01) (Figure 7B) compared to co-transfection with the negative control mimic. These results supported the hypothesis that miR-34c-3p directly targeted the PLCXD3 3′-UTR. Moreover, the western blots suggested that miR-34c-3p repressed the expression of PLCXD3 at the protein level (Figure 8). The signals from PLCXD3 immunostaining were evaluated using qualitative immunohistochemistry to determine PLCXD3 expression in patients with SO and OA. The PLCXD3 protein is expressed at high levels in spermatids of an OA patient's testis, but there was no immunostaining in the SO patient. Therefore, altered PLCXD3 gene expression may be a biomarker for men with idiopathic SO (Figure 7C).

## DISCUSSION

Despite the advances in assisted reproductive technologies (ART), infertility is a major health problem worldwide. It is necessary to determine the underlying genetic basis of male factor infertility to prevent abnormal genetic messages from being passed through ART and resulting in serious implications for the developing zygote. However, a large proportion of infertile males do not receive a clear diagnosis and are reported as idiopathic or unexplained, reflecting our poor understanding of the basic mechanisms regulating spermatogenesis and sperm function. To date, the most common genetic defects are structural and numerical chromosome abnormalities that are diagnosed in up to 14% and 10% of azoospermic and oligozoospermic patients, respectively [18]. Recent studies have shown that miRNAs play an important role in gene expression for many biological processes. Therefore, it is crucial to identify the target mRNAs to understand the biological function of miRNAs. At present, the lack of knowledge regarding *bona fide* miRNA target genes hampers a full understanding of the biological functions that are deregulated by aberrant miRNA expression. We adopted a strategy that integrated miRNA and mRNA profiles of testes from SO and OA patients (normal spermatogenesis in histology) to overcome this limitation. First, we identified the significantly up/downregulated microRNAs and mRNAs between the two groups. Second, we used the microarray expression data and online databases to identify thousands of highly correlated miRNA-mRNA pairs. Third, we validated the potential interactions and prediction results based on the potential mRNA targets selected. The evidence showed that this strategy significantly decreased the false-positive rate of the identification of the mRNA targets of miRNAs. The combination of expression profiling and computational predictions is an effective method for discovering miRNA-mRNA interactions.

miR-34c was observed in the late stages of meiosis (pachytene spermatocytes and round spermatids) and is likely to influence the germinal phenotype [19, 20]. The downregulation of miR-34c in our microarray results may affect germinal lineage differentiation, which closely correlates with the low sperm concentration in SO disease. Recently, miR-34c was reported to regulate a number of targets, including Nanos2 [20], p53 [21] and RARg [22], which are mostly involved in the biological process of male germ cell differentiation. In this study, we observed an inverse relationship between the expression of miR-34c-3p and PLCXD3 in SO patients. Furthermore, the luciferase activity and the level of the PLCXD3 protein were downregulated by the miR-34c-3p mimic. This finding suggests that PLCXD3 is a target of miR-34c-3p. PLCXD3 is a novel PI-PLC subtype that only contains the characteristic X-domain. To date, there is very little information regarding the functions of these proteins. The preliminary results from our experiments showed that PLCXD3 exhibited tissue-specific expression profiles in mice and humans. Immunocytochemistry also revealed the expression of PLCXD3 at the late stages of spermatogenesis, from late to maturing spermatids. Taken together, these data suggest that this novel protein may exert fundamental and as yet uncharacterized roles in cell physiology. It is of great clinical importance to identify more accurate biomarkers than hormones, inhibin B, etc., that can indicate the presence or absence of spermatogenic foci in the testis. Therefore, altered PLCXD3 gene expression may be a marker for men with idiopathic NOA and SO.

The new differentially expressed mRNA and miRNA candidates identified in this study can be used to further characterize miRNA-mRNA interactions and their functions in male infertility. Several of the miRNAs (miR-1246 and miR-630) and genes (SLC1A3, PRKAR2B, and PRDX1) identified in our analysis have also been addressed in previous studies. However, we identified a large number of novel miRNAs (miR-411-5p, miR-375, miR-410, and miR-758) and genes (HYDIN, WDR65, PAQR5, MGARP, and FLJ45983) that have not previously been detected and may have roles during the steps of spermatogenesis. Although further functional evidence is also required, these results provide insights into the pathoetiology of SO, as well as reproductive fitness at the molecular level, and suggest a series of biomarkers or targets for therapy. Moreover, our integrated data analysis strategy revealed that multiple pathways were deregulated in spermatogenesis, such as spermatid development, cell maturation, axonemal dynein complex assembly and the Jak-STAT signaling pathway, which agrees with previous studies implicating these pathways in SO. These findings indicated that the pathologic mechanisms of SO are significantly different from normozoospermic men with obstructive-azoospermia. In most cases of OA, normal or near-normal sperm production continues in the testis, although the excurrent duct is obstructed. The difference in the developmental trajectories and molecular changes may contribute to the difference in phenotypes between SO and OA.

## CONCLUSIONS

In conclusion, this study is the first to identify the involvement of miRNAs and mRNAs in the regulation of spermatogenesis using testis biopsies of patients with severe oligozoospermia and obstructive azoospermia. Differentially expressed mRNAs and miRNAs were identified, and their predicted interactions and GO/KEGG pathways were analyzed. Both the miRNAs and targeted genes discovered in this study are resources that can be used to understand the molecular mechanisms of severe oligozoospermia. Our results are the first to indicate that PLCXD3 was regulated by miR-34c-3p and played an important role in testicular failure. However, the functional relevance of the aberrant expression of the identified genes in men with low sperm counts warrants further investigation.

## MATERIALS AND METHODS

### Patients

Testicular biopsy specimens were obtained from 3 patients (aged from 23 to 37 years) with SO and from 3 patients (aged from 25 to 28 years) with OA (see Supplemental File 1: Patient information) for the microarray analysis. These patients underwent testicular sperm extraction (TESE) for assisted reproduction and/or diagnostic biopsy for histological examination. For the histological evaluation, the specimens were stained with hematoxylin and eosin (H&E) and analyzed by microscopy. SO was defined as a reduced number of sperm in the male ejaculate or less than 5 million sperm per milliliter. OA was defined as follows: (1) motile spermatozoa were sampled from a microsurgical epididymal sperm aspiration (MESA) or (2) a considerable number of mature spermatozoa were sampled from TESE. An ideal study population of normal controls would consist of volunteers with known fertility, but the difficulties in acquiring testicular samples makes this strategy impractical. Instead, samples from urology patients who had no history of meiotic defects or infertility and exhibited normal spermatogenesis upon histological examination were analyzed. In addition, none of the controls were exposed to adjuvant hormonal therapy prior to orchiectomy. The infertile male patients who visited Xiamen University Affiliated First Hospital received a routine semen examination according to the 2010 WHO criteria. The levels of serum follicle-stimulating hormone (FSH), luteinizing hormone (LH), and total testosterone were measured before the operation. The ethics committees of Xiamen University Affiliated First Hospital (Institutional Review Board Number: KYX-2015-001) approved the study protocols, and each participant provided written informed consent. The methods were performed in accordance with the approved guidelines.

### RNA extraction

Immediately after retrieval, the testicular tissues were stored at -80°C until further processing. Total RNA, including miRNAs, was isolated using the miRNeasy Micro Kit (Catalog no. 217084, Qiagen, Germany), according to the manufacturer's protocol. On-column DNase digestion was performed. The RNA concentration, purity (including the detection of DNA contamination) and RNA integrity number (RIN) were determined using a NanoDrop ND-1000 spectrophotometer (Peqlab, Erlangen, Germany), an Agilent 2100 Bioanalyzer and an RNA 6000 NanoLabChip Kit (Agilent Technologies), respectively. The inclusion criteria required a minimum RIN of ≥7.0 for the sample to be acceptable for microarray analysis (see Supplemental File 2: Electropherogram of RNA). Each RNA sample was then divided into two aliquots that were used for either the miRNA microarray or the gene expression microarray. The RNA used for the tissue expression patterns was extracted from various mouse tissues using TRIzol reagent (Invitrogen), according to the manufacturer's protocol.

### Gene expression microarray

For microarray hybridization, 100 ng of total RNA were labeled using the Low Input Quick Amp Labeling kit (Agilent 5190-2305) according to the manufacturer's instructions and were then hybridized to the Agilent SurePrint G3 Human gene expression 8×60K microarray according to the manufacturer's protocol. Briefly, 100 ng of total RNA was converted to cDNAs, followed by *in vitro* transcription and incorporation of Cy3-CTP into the nascent cRNAs. After fragmentation, the labelled cRNAs were hybridized to the microarrays for 17 h at 65°C and scanned as described in the manufacturer's protocol. The arrays were washed and scanned using an Agilent G2565CA microarray scanner at a photomultiplier tube (PMT) value of 100% and 5 μm resolution. The intensity data were extracted using the Feature Extraction 10.7.3.1 Software (Agilent Technologies).

### miRNA microarray

Total RNA samples were spiked using the MicroRNA Spike-In Kit (Agilent Technologies) to assess the labeling and hybridization efficiencies. After the spiked total RNA was treated with alkaline calf intestine phosphatase, a labeling reaction was initiated with 100 ng of total RNA per sample. T4 RNA ligase, which incorporates cyanine 3-cytidine biphosphate (miRNA Complete Labeling and Hyb Kit; Agilent Technologies), was used to label the dephosphorylated RNA. The cyanine 3-labeled miRNA samples were subsequently prepared for one-color hybridization (miRNA Complete Labeling and Hyb Kit). The labeled miRNA samples were hybridized to the Agilent Human miRNA Microarray 8×60K Release 16.0 (Release 16.0, 8×60K format; Agilent Technologies) for 20 h at 55°C. After washing the microarray slides with buffers with increasing stringency (Gene Expression Wash Buffers; Agilent Technologies), the slides were dried with acetonitrile. Fluorescent signal intensities were detected on an Agilent Microarray Scanner (Agilent Technologies) with the Scan Control A.8.4.1 Software (Agilent Technologies) and were extracted from the images using the Feature Extraction 10.7.3.1 Software (Agilent Technologies). All of the steps described above were performed according to the manufacturer's instructions.

### Microarray data analysis

The raw gene expression and miRNAs microarray data were normalized using the GeneSpring GX software version 12.0 (Agilent Technologies). The signal values were transformed to the log base 2, and then quantile and percentile shift was applied to obtain an equal distribution of probe signal intensities. The comparative analysis of the SO and control group samples was performed using the *t*-test (p-values) and SAM (http://www-stat.stanford.edu/~tibs/SAM/). Compared with the expression level of the reference RNA, the genes and miRNAs were described as differentially expressed if the p-values were < 0.05, and the fold change (FC) was greater than 2 or less than 1/2.

### GO and KEGG pathway analyses

Gene Ontology (GO) and KEGG pathway analyses were performed using Cytoscape V2.7 (http://cytoscape.org/) with the ClueGo V1.3 plug-in [11]. ClueGo determines the distribution of the target gene list across the GO terms and pathways. The p-value was calculated using a right-side hypergeometric test, and the Benjamini-Hochberg adjustment was used to correct for multiple tests. An adjusted p-value of < 0.05 indicates a statistically significant deviation from the expected distribution, and the corresponding GO terms and pathways were enriched in the target genes. We analyzed all of the differentially expressed genes and the target genes of the deregulated miRNAs using the GO and KEGG pathway analyses.

### miRNA-mRNA enrichment analysis and negative correlation

The putative target gene sets of the differentially expressed miRNAs were predicted using the following algorithms: MiRanda [23], Pictar [24], TargetScans [25], Microrna.org [26], PITA [27], MicroT [28], and RNA22 [29]. The p-values were calculated using a right-side hypergeometric tests, and the Benjamini-Hochberg adjustment was used to correct for multiple tests. An adjusted *p*-value of < 0.05 indicates a statistically significant deviation from the expected distribution. Pearson's correlation coefficients between the miRNA and genes with the top 75% variance were computed using R (http://www.R-project.org) to determine whether the expression levels of each miRNA and its mRNA targets were negatively correlated (correlation < 0 and Fisher P corrected by the FDR (False Discovery Rate) < 0.05). For each miRNA-target, we designed a variable theta (*θ*) to represent the strength of the relationship. *θ* = FC1/FC2, FC1 indicates the fold change in the significantly differentially expressed genes, and FC2 indicates the fold change in the significantly differentially expressed miRNAs. If FC < 0, then the reciprocal is used.

### Functional network analysis

First, TarBase [30] was used to compare the experimentally validated miRNA targets with the putative targets. Second, the human protein-protein interaction networks (PPIN) were downloaded from the Human Protein Reference Database [31]. Finally, the igraph package of the statistical language R was used to generate functional profiles. The network visualization and analysis tool Cytoscape [32] was used to identify the putative target genes for the predicted miRNAs in SO within PPIN. The Markov cluster algorithm [33] was used to identify the highly connected modules within the global networks.

### Hierarchical clustering

Hierarchical clustering was performed in Cluster version 3.0 and Java TreeView version 1.1.6 to identify and visualize the patterns within the microarray dataset. Using clustering algorithms, the samples and probes were grouped based on the similarities in the expression profiles. The probe set was filtered based on standard deviation to exclude the probes with the least variance. Average linkage and median centering were the chosen parameters. Both unsupervised and supervised clustering were used. In the unsupervised method, all genes were included, whereas supervised clustering only used the significant genes as input.

### Reverse transcription quantitative real-time PCR (RT-qPCR) for miRNAs

Reverse transcription quantitative real-time PCR was performed to confirm the array results using the miScript PCR System and the 10×miScript Primer Assays for hsa-miR-375, hsa-miR-1246, hsa-miR-410 and hsa-miR-758 (Qiagen). Each 10 μL reaction consisted of 1 μL of cDNA, 2 μL of RNase-free water, 1 μL of 10× miScript primer assay, 1 μL of 10× miScript universal primer, and 5 μL of 2× QuantiTect SYBR Green PCR master mix. The cycling conditions started with initial activation step at 95°C for 15 min, then 40 cycles of three steps each (94°C for 15 s, 55°C for 30 s, and 70°C for 30 s), and then a dissociation curve was constructed to verify the specificity of the PCR products. The RNU6B snRNA primer assay (Qiagen) was chosen as an endogenous reference for normalization, and all procedures were performed according to the manufacturer's recommendations.

### RT-qPCR for gene expression

Specific qPCR primers were designed for 7 mRNA genes (PLCXD3, SLC1A3, PRKAR2B, HYDIN, WDR65, PRDX1 and ADAMTS5) that were differentially expressed in the discovery screen to validate the different expression levels of the mRNA genes determined by microarray. GAPDH was selected as the internal control. Two micrograms of total RNA from each sample were reverse transcribed into cDNAs using a Quanti-Tect Reverse Transcription Kit (Qiagen), according to the manufacturer's protocol. The reverse transcription products were amplified using the TransStar Top Green qPCR Super Mix (TransGen, Beijing, China), according to the manufacturer's protocol. The reaction volume was 20 μL and included 2 μL each of the 5 mM Forward and Reverse primer stocks, 10 μL of 2× TransStart Green qPCR SuperMix, 2 μL of the cDNA templates, 0.5 μL of Passive Reference Dye I, and 3.5 μL of ddH2O. The thermal cycling profile was as follows: 94°C for 30 s; 40 cycles of 94°C for 5 s, 55°C for 30 s, and 72°C for 30 s; followed by 95°C for 60 s, 55°C for 30 min, and a final incubation at 95°C for 30 s.

### Cell culture

GC-1 cells (mouse-derived spermatogonia line), TM-4 cells (mouse-derived Sertoli cell line), NCM460 cells (human colon cell line) and HEK-293T cells (human embryonic kidney cell line) were cultured in fresh Dulbecco's modified Eagle's medium supplemented with 10% fetal bovine serum in 5% CO2 incubators at 37°C. Adherent cells were passaged every 1-2 days with 0.5 mg/ml trypsin (1:250) and 0.53 mM EDTA.

### Plasmid construction and transfection

The 3′-UTR of PLCXD3 was amplified from the cDNA of the SO patient by PCR using the following custom primers: forward 5′-TTCCATGAATAAGATGGAGAAAGCTCATTG-3′ and reverse 5′-GTACCTTACCATTTACTGGGTACTTGCA-3′. The pmirGLO Dual-Luciferase miRNA Target Expression Vector (Promega, USA) was used to confirm the function of the putative miR-34c-3p binding site in the 3′-UTR of PLCXD3. Cells were washed once with serum-free medium and were then incubated with 4 mL of serum-free medium for 4-6 hours. The miRNA (or miRNA inhibitor and scrambled miRNA) and Lipofectamine 2000 transfection reagent (Life Technologies, USA) were separately mixed with 500 μL of Opti-MEM I Reduced Serum Medium (Gibco Life Technologies, Grand Island, NY) for 15 minutes. Then, the two mixtures were combined and incubated for 20 minutes at room temperature. The Lipofectamine 2000-miRNA mixture was added to the cells and incubated at 37 °C for 12 hours. Subsequently, 5 mL of fresh medium containing 10% fetal bovine serum were added to the flasks and the cells were maintained in culture until the experiments were conducted.

### Dual luciferase reporter gene assay

HEK-293T cells were divided into groups according to the concentration of the transfected plasmid. The 293T cells were seeded in 48-well plates in triplicate, allowed to settle for 24 hours, and then co-transfected with different concentrations of plasmids using Lipofectamine 2000 (Invitrogen, Carlsbad, CA). Luciferase and Renilla activities were measured 48 hours after transfection using the Dual-Luciferase Reporter Assay Kit (Promega Corporation, Madison, WI), according to the manufacturer' s instructions.

### Immunoblotting

The cells (GC-1, TM4 and NCM460 cells) were transfected with the indicated miRNA mimics and inhibitors (hsa-miR-34c-3p mimic: 5′-aaucacuaaccacacggccagg-3′, hsa-miR-34c-3p mimic inhibitor: 5′-ccuggccgugugguuagugauu-3′; negative control mimic: 5′-uuuguacuacacaaaaguacug-3′; negative control inhibitor: 5′-caguacuuuuguguaguacaaa-3′). Forty-eight hours later, the cells were lysed in RIPA buffer (Norgen-Biotek Corp.) containing 1× Halt Protease Inhibitor Cocktail (Pierce Inc., Rockford, IL, USA). Forty micrograms of total protein were separated and blotted using the Bio-Rad V3 Western work flow system, according to the manufacturer's recommendation. Immunoblotting was conducted by incubating the membrane with an anti-PLCXD3 rabbit polyclonal antibody (1:500, Santa Cruz Biotechnology, sc-137672) overnight at 4°C. A horseradish peroxidase (HRP)-conjugated goat anti-rabbit antibody (1:3,000, Cell Signaling, 7074) was used as the secondary antibody, whereas an HRP-conjugated anti-β-tubulin antibody (1:10,000, Abcam, ab6046) was used as the loading control.

### Immunohistochemistry

The tissues were collected and fixed overnight in 4% paraformaldehyde. Then, they were dehydrated in a graded ethanol series, cleared in a xylene solution, and then embedded in paraffin wax. The paraffin-embedded testes were serially sectioned at 5 μm. The sections were microwaved in the antigen unmasking solution, incubated in methanol/hydrogen peroxide, blocked with a bovine serum albumin solution, and incubated with a rabbit polyclonal anti-PLCXD3 antibody (1:200, Atlas, HPA046849) overnight at 4°C. After washing with PBS (3 × 10 minutes), the sections were incubated with diaminobenzidine.

### Statistical analysis

All the results for continuous variables are expressed as the mean ± SD, and the results for the categorical variables are expressed as the number of cases and percentage. The significance of the differences between the groups was assessed using Student's *t*-test or 1-way analysis of variance for the continuous variables and the χ^2^ test for the categorical variables. All tests were 2-tailed, and a P-value of 0.05 was considered to indicate significance. The data were analyzed with SPSS for Windows version 12.0 (SPSS Inc., Chicago, IL).

## SUPPLEMENTARY MATERIALS FIGURES AND TABLE


