# Supplementary material for: Integrated analysis miRNA and mRNA profiling in patients with severe oligozoospermia reveals miR-34c-3p downregulates PLCXD3 expression
Source: Oncotarget. 2016 Jul 29;7(33):52781–96. doi: 10.18632/oncotarget.10947 (PMC5288148; doi:10.18632/oncotarget.10947)
Supplement: Supplementary file 1 [file oncotarget-07-52781-s001.pdf]

Integrated analysis miRNA and mRNA profiling in patients with severe oligozoospermia reveals miR-34c-3p downregulates PLCXD3 expression

Supplementary Material

Supplemental table 1. Patient hormone, semen and histology characteristics

Group	No.	Sperm found via testicular extraction		FSH (IU/L)	LH (IU/L)	Testosterone (ng/dL)	Elastase (ng/mL)	Neutral α-glucosidase (mU/mL)	Fructose (mg/dL)	Histology
		Mature	Mobile							
SO	1	Yes	Yes	12.78	14.32	400.42	654.27	13.11	146.72	Seminiferous tubules have thickened basement membrane and hyperplastic Sertoli cells and Leydig cells. The spermatogenetic cells of different stages show moderate decrease, especially mature spermatid. There are some distribution of collapsed tubules.
	2	Yes	Yes	13.12	15.87	419.98	578.54	15.28	145.29	
	3	Yes	Yes	13.90	15.63	411.12	593.54	14.73	156.17	
OA	1	Yes	Yes	8.23	9.76	399.21	178.79	25.67	289.35	Seminiferous tubules with evidence of spermatogenesis. Uniform distribution of non-collapsed tubules with normal appearance.
	2	Yes	Yes	8.56	9.45	399.19	173.23	26.45	280.43	
	3	Yes	Yes	7.23	9.98	400.78	188.31	29.90	290.48	

SO: severe oligozoospermia, OA: obstructive azoospermia, FSH: follicle-stimulating hormone, LH：luteinizing hormone.

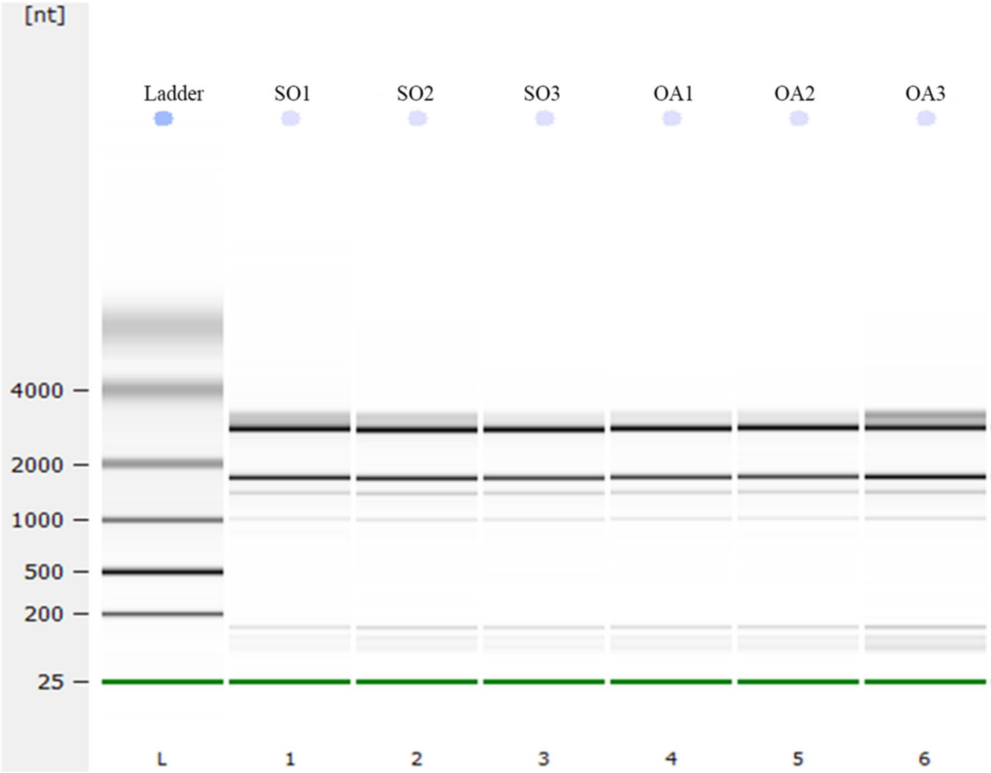

Electropherogram of RNA

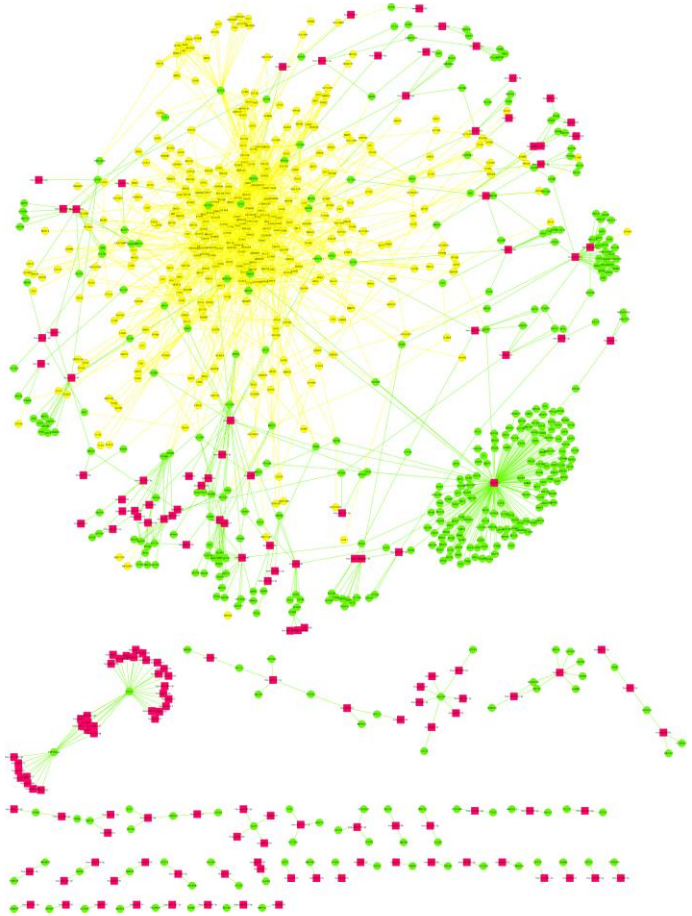

network
